# Supplementary material for: Human Serum Albumin Nanoparticles: Synthesis, Optimization and Immobilization with Antituberculosis Drugs
Source: Polymers (Basel). 2023 Jun 22;15(13):2774. doi: 10.3390/polym15132774 (PMC10347201; doi:10.3390/polym15132774)
Supplement: Supplementary file 1 [file polymers-15-02774-s001.zip › polymers-2431018-supplementary.pdf]

Supporting Information

Human serum albumin nanoparticles: synthesis, optimization and immobilization with antituberculosis drugs

Aldana Galiyeva*, Arailym Daribay, Tolkyn Zhumagaliyeva, Lyazzat Zhaparova, Daniyar Sadyrbekov, Yerkeblan Tazhbayev*

To study the degree of release from the polymer nanoparticles, the amount of drug released was recorded by HPLC. The simultaneous determination of rifampicin and isoniazid was done using the combined methods of [1] and [2].

We determined isoniazid and rifampicin simultaneously by HPLC. HPLC chromatograms show separation for isoniazid and rifampicin (Figure 1). Each drug leaves the HPLC column at a specific time.

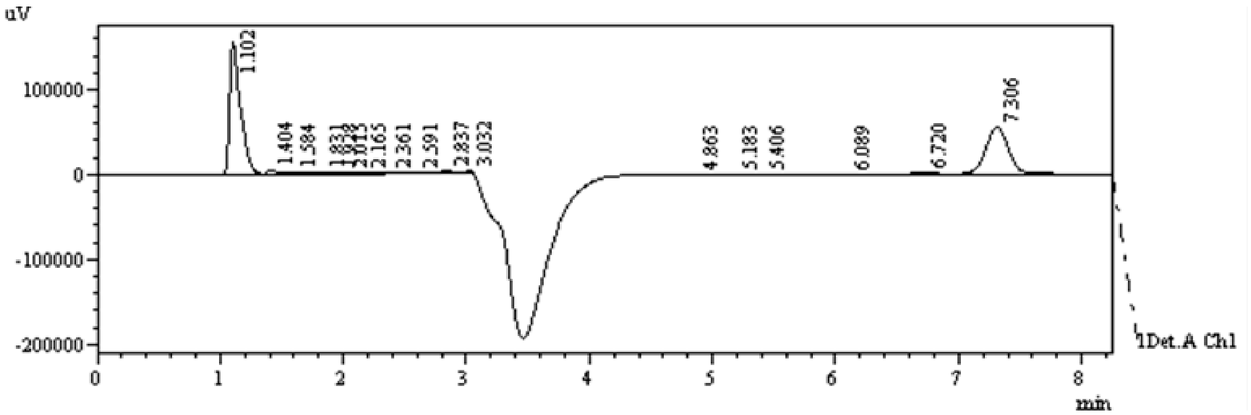

Figure S1. HPLC chromatogram showing the separation of isoniazid (at 1.102 min) and rifampicin (at 7.306 min)

References:

1. https://sielc.com/hplc-determination-of-rifampicin

2. T. T. Mariappan, Baljinder Singh, Saranjit Singh. A Validated Reversed-Phase (C18) HPLC Method for Simultaneous Determination of Rifampicin, Isoniazid and Pyrazinamide in USP Dissolution Medium and Simulated Gastric Fluid // Pharmacy and Pharmacology Communications. – 2000. – Vol. 6, Issue 8. – Pp. 345-349 https://doi.org/10.1211/146080800128736187
